# Impact of Inaccurate Clinical Coding on Financial Outcome: A Study in a local hospital in Najran, Saudi Arabia.

**DOI:** 10.12688/f1000research.149154.1

**Published:** 2024-07-22

**Authors:** Salem Albagmi

**Affiliations:** 1 Health Sciences, Prince Sultan Military College of Health Sciences, Dammam, 0000, Saudi Arabia

**Keywords:** Clinical Coding, Diagnosis, Hospitals

## Abstract

**Background:**

Coding in medical procedures is crucial for patients, and errors made by hospital administration during the coding process can have an impact on both the financial results and the course of therapy. The present study aims to assess the accuracy of diagnostic and procedural codes as recorded by the hospital’s coders and to also evaluate their impact on the hospital’s revenue.

**Methods:**

In a local hospital in Najran, Saudi Arabia, a cross-sectional observational analysis was conducted on patients with a clinical coder. The percentage of precision and error following the re-coding of cases was calculated using a statistical analysis.

**Results:**

Primary diagnosis was incorrectly coded in 57 (26 per cent) records, and secondary diagnosis was incorrectly coded in 21 (9.9 per cent) records. Inaccurate medical labelling has been seen in emergency rooms, operating rooms, and gynaecology facilities.

**Discussion:**

The percentage of records with the most incorrect coding was found to be 16 (7.5 per cent) in the emergency room, 10 (4.7 per cent) in the surgical clinic, and 5 (2.3 per cent) in the gynaecology/OBS clinic. Six (2.8 per cent) records in the private clinic had inaccurate secondary diagnoses, followed by four (1.9 per cent) and two (1 per cent) records in nephrology.

**Conclusion:**

The percentage of inaccurate clinical codes in primary diagnoses reached (26.8 per cent) and the percentage of incorrect clinical codes in secondary diagnoses reached (9.9 per cent).

## Introduction

Clinical coders transform clinical terminologies into coded language, which represents a system of figures and letters. Diagnostic and procedural codes are major components of the coding process. In Saudi Arabia, clinical coders use the International Statistical Classification of Diseases and Related Health Problems, Tenth Revision, Australian Modification (ICD-10-AM) for illnesses and health issues, and the Australian Classification of Health Interventions (ACHI) for procedures. ^1^ These two systems aid medical professionals in the creation of a standardized coding for illnesses, procedures, medical consultations, and causative factors for accidents. Moreover, hospital reimbursement, audits, studies, planning, and administration also rely on coded data. ^2^

The cost of care and income are calculated using two different financial systems i.e. Diagnostic Related Group (DRG) and Case Mix Index (CMI). DRG, developed in the United States, was then adopted by other nations. Its main purpose is to collect data on the patients treated, and the amount earned by the hospital. With the assignment of coding as per the International Classification of Diseases, Ninth Revision, and Clinical Modification (ICD-9-CM) to the illness and procedures, DRG categorizes the patients based on their diagnosis and thus monitors the fact that all the patients are charged the same amount for a particular procedure. The Centers for Medicare and Medicaid Services (CMS) implemented the Inpatient Prospective Payment System, based on DRG. ^1^^,^^2^

CMI classifies patients according to their clinical characteristics and acts as a provider payment tool that helps monitor hospital resources and income. Despite the original principle for its development was the calculation of hospital payments in terms of patient care, it has recently gained momentum as a platform for public reporting of quality indicators and costs for disease severity (Indicator or costs ÷ CMI of the medical centre). This helps in making comparisons among various medical centres. However, the authenticity of CMI may be affected by the accuracy of the documentation skills of the physician and the coding expertise of the coder using ICD-9-CM codes. ^3^

Accuracy in the coding system is very crucial as the entry of a single wrong code can result in a domino effect which might end in affecting the hospital’s income. Thus, the accuracy of clinical codes which are eventually used for payment, health statistics, research, and planning purposes, requires thorough verification. ^4^^,^^5^ The present study aimed to assess the accuracy of diagnostic and procedural codes as recorded by the hospital’s coders and to also evaluate their impact on the hospital’s revenue.

## Methods

The study was reviewed by the institutional review board which provided an exemption due to the retrospective nature of the study. The coded cases, from a local tertiary care hospital in Najran, Saudi Arabia, with complete patient medical records consisting of diagnosis, procedures done, and the bills paid (either by the patient or the insurance company) were included in the study on a non-probability consecutive basis. The coding was done using ICD-10-AM, and ACHI.

The reviewing process, done by the HIM department staff, involved all the health records as well as the inpatient fact sheet (part of the health record) that is used by clinical coders to write diagnoses and procedural codes. The recoding was done using the ICD-10-AM, and ACHI which was the same as used by the hospital’s coders. Following the reviewing and recoding processes, the differences were documented in a special form and then the codes were compared to evaluate the accuracy of the initial codes. The comparison also comprised an assessment of the change in the total charges incurred based on the change in the code (if any).

### Ethical statement

The approval was obtained from the Institutional Review Board of Prince Sultan Military College of Health Sciences (IRB-2021-HIM-023) on June 6, 2023. The study was conducted in accordance with the declaration of Helsinki.

### Data analysis

The inaccuracy observed during the reviewing and recoding process was determined using the following formula:

$$\;\text{Accuracy}\;\left(\%\right)=\frac{\text{Inaccurate codes in diagnosis}\times 100}{\text{Total}\;\mathrm{no}.\mathrm{od}\;\text{coded cases}}$$

Furthermore, the total financial impact that might have been incurred due to inaccurate coding and represented the total inaccurate claims was calculated by the formula given below:

Total Financial Impact = Charges before the re-coding process - Total charges of all cases after the recoding process

### Total financial impact

The chi-square test was used to assess an association between errors during the coding process and the diagnosis made. Cohen’s Kappa test was used to examine the degree of agreement between the assignment of every code using DRG ( https://sourceforge.net/projects/drg-project/) before (by the hospital coders) and after the recoding process (by Health Information Management department staff). Below are mentioned the Kappa values and their interpretations:

• 0.01-0.20 = Low agreement
• 0.21-0.40 = Reasonable agreement
• 0.41-0.60 = Moderate agreement
• 0.61-0.80 = Considerable agreement
• 0.81-1.00 = Excellent agreement

All data analyses were done using IBM SPSS version 20.0 ( https://www.ibm.com/support/pages/spss-statistics-260-fix-pack-1), and a p-value <0.05 was deemed significant.

## Results

**Figure 1.**
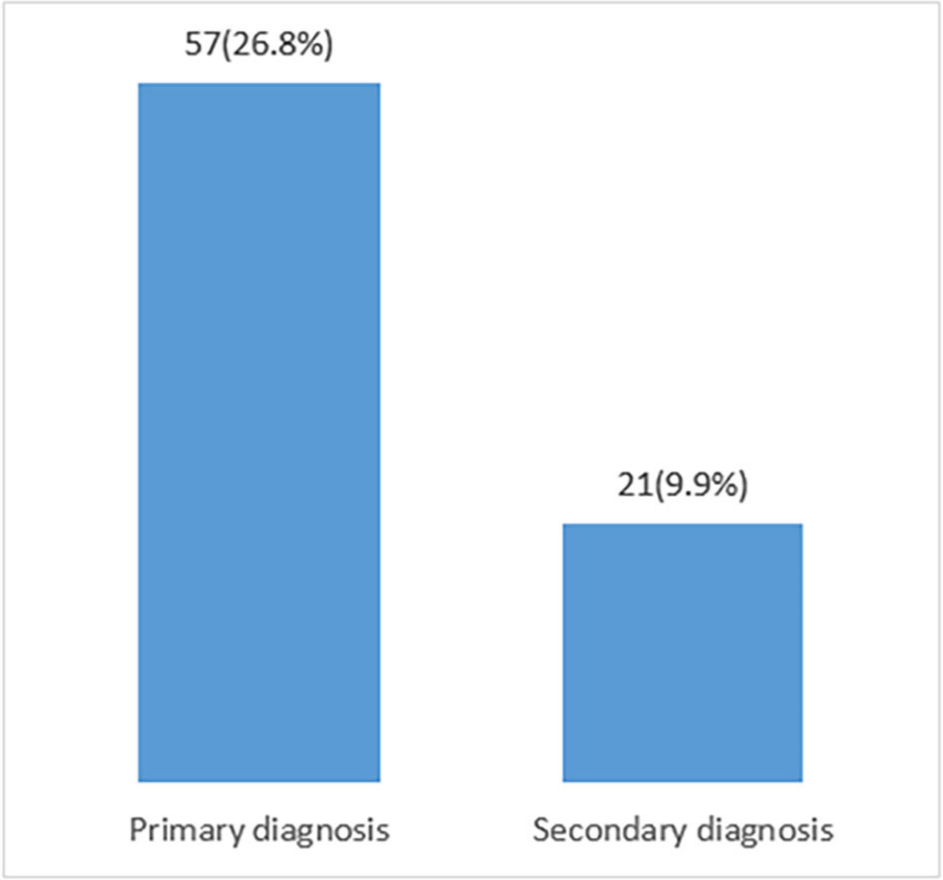
Percentage of inaccurate medical codes.

Inaccurate coding of primary diagnosis was observed in 57 (26.8 per cent) records, and secondary diagnosis was observed in 21 (9.9 per cent) records ( Figure 1).

Incorrect medical coding was most commonly seen in emergency departments, operating rooms, and obstetrics and gynaecology facilities. The Emergency Room had the greatest incidence of incorrect coding at 7.5 per cent, followed by the Surgery Clinic at 4.7 per cent and the Gynecology and Obstetrics Clinic at 2.3 per cent ( Table 1). Six (2.8 per cent) incorrect secondary diagnostic documents were found in the examining clinic, four (1.9 per cent) in the nephrology department, and two (1.1 per cent) in the diabetes department. There was only modest agreement between the clinician coder and the staff coder on the primary diagnosis, and only low agreement on the secondary diagnosis with a kappa score of 0.12 (kappa score of 0.48).

**Table 1.** Error in clinical coding due to DRG implementation.

Variables	Clinics	Inaccurate cases	p-value
Primary diagnosis	Emergency room	16 (7.5%)	<0.05
	Surgery clinics	10 (4.7%)	
	Gynae/OBS	5 (2.3%)	
Secondary diagnosis	Consulting clinic	6 (2.8%)	0.05
	Nephrology	4 (1.9%)	
	Diabetes	2 (1%)	
Types of severity level	I	11 (19.3%)	<0.001
	II	18 (31.6%)	
	III	28 (49.1%)	

### Inaccurate codes & financial consequences

**Figure 2.**
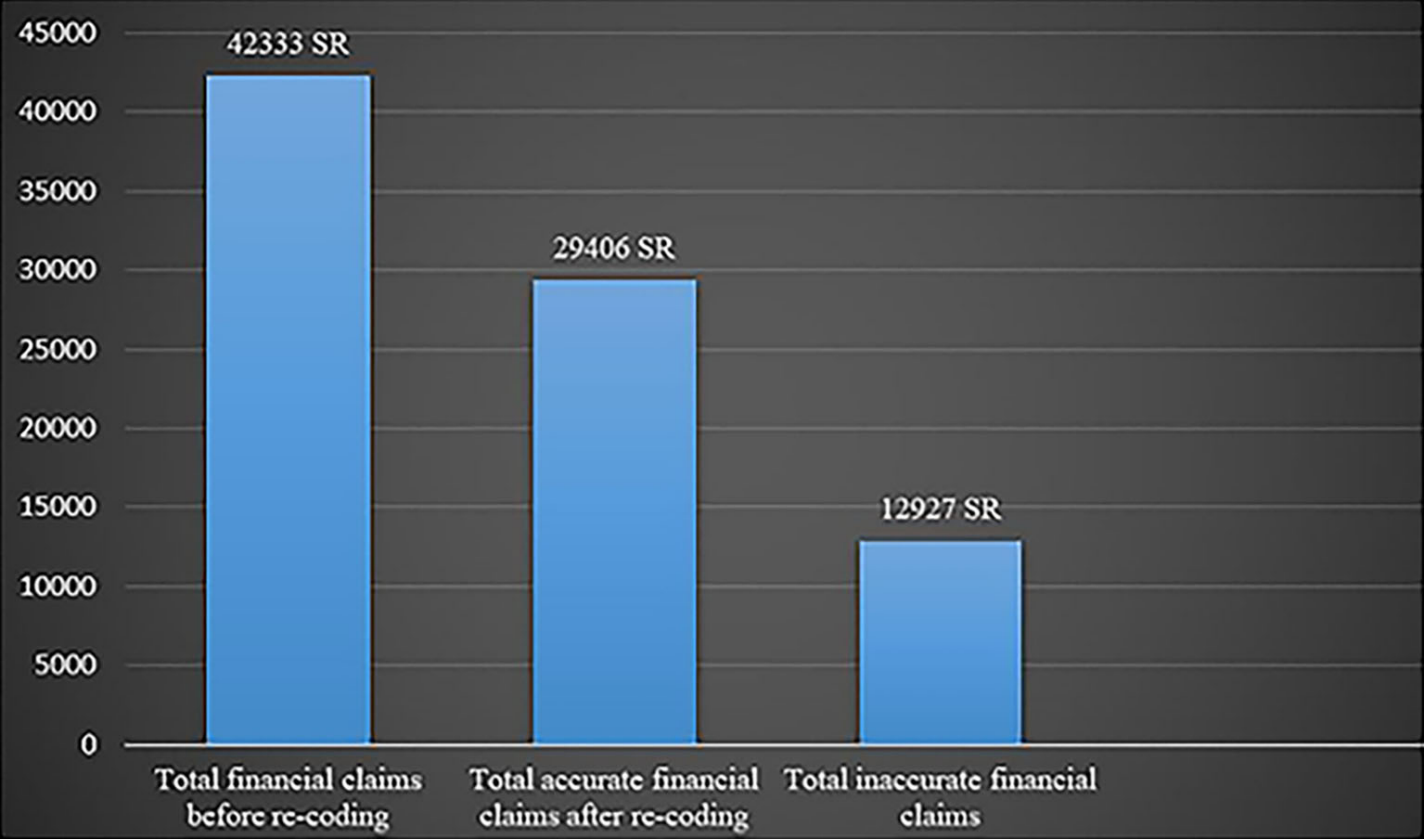
Financial claims before and after the re-coding process.

Financial claims before and after re-coding are shown in Figure 2. Because of financial differences caused by inaccurate medical codes for primary and secondary illnesses, certain financial claims have been denied by insurance companies. Before the re-coding process of this research, a total of 42,333 SR (11287.46 $) was in the cash claims, the re-coding procedure was reduced to 29,406 SR (7840.67 $) in total in terms of proper financial claims, incorrect medical coding caused an error of 12,927 SR (3446.79 $) in payment claims.

## Discussion

The process of assigning ICD codes is complicated due to the numerous steps and the participants involved in the process leading to the creation of opportunities for error. Evaluating the accuracy of the code will subsequently improve healthcare decisions. ^6^ Clinical codes are considered wrong or inaccurate when the clinical coder’s codes differ from the independent coding auditor’s code. ^7^ A wrong code for the disease or procedure leads to assigning a wrong DRG code, which eventually will generate an incorrect amount. ^8^ The issue of inaccurate medical coding as observed in the present study and its financial ramifications, not only impacted hospitals’ financial claims but also had a profound effect on the treatment procedure of the patients. ^9^

The present study reported approximately one-third (36.7 per cent) of the samples were incorrectly recorded in primary and secondary diagnosis, which resulted in the hospital being liable for 12,927 Saudi Riyals (3446.79 $) in terms of lost revenue. The findings found concordance with studies conducted by Zafirah et al., ^1^ and Jordan et al. ^4^ in the service of liaison psychiatry, and plastic surgery cases. According to the findings of Jordan et al., ^4^ 12.7 per cent of patients switched to a higher-paying DRG and the hospital gained €305,349 in reimbursement. Hospitals might lose up to £29,000 if patients are categorized incorrectly, according to Mole et al. ^5^

One of the reasons for the commission of errors by the coders is their being of non-medical background. The hospital coders rely on accurate documentation in patient notes, including admission documents, operating notes, and discharge summaries. Hospital coders cannot retrieve information from other sources such as old notes and letters for important information such as medical comorbidities and medications. Therefore, complete and accurate medical clerking by the admitting team is essential for hospital coders to accurately record comorbidities required for HRG tariffs and to reduce errors. ^9^

During the present study, various problems were encountered which created hurdles in the process of sample collection. The COVID-19 pandemic restricted access to researchers. Hybrid health records could not gather information electronically and the process of data collection and processing was time-consuming. ^10^ The study failed to have access to procedure codes and documentation during sample collection because of confidentiality issues. Furthermore, private hospitals are also reluctant to produce their financial statements and may attempt to conceal data for fear of exposing financial discrepancies claimed by patients or insurance companies due to their faults.

### Ethical considerations and consent

Before conducting this research, the researcher obtained the approval from Institutional Review Board of Prince Sultan Military College of Health Sciences (IRB-2021-HIM-023) on June 6, 2023. The study was conducted in accordance with the declaration of Helsinki.

The consent from the participants was waived by the ethical approval committee.

## Data Availability

FigShare Research Samples Impact of Inaccurate Clinical Coding on Financial Outcome A Study in a local hospital in Najran, Saudi Arabia.xlsx. [ https://doi.org/10.6084/m9.figshare.25908010.v2]. This project contains the following underlying data:
1. Data file
