# Supplementary material for: Impact of Inaccurate Clinical Coding on Financial Outcome: A Study in a local hospital in Najran, Saudi Arabia
Source: F1000Res. 2024 Jul 22;13:820. [Version 1] doi: 10.12688/f1000research.149154.1 (PMC11342027; doi:10.12688/f1000research.149154.1)
Supplement: Peer review history

# Reviewer report: Reviewer response for version 1

**DOI:** 10.5256/f1000research.163572.r314504

**Published:** 2024-08-22

**Authors:** Muteb Alshammari

**Affiliations:** 1 Department of Health Informatics, College of Public Health and Health Informatics, University of Hail, Hail, Hail Province, Saudi Arabia

The manuscript effectively addresses the importance of accurate clinical coding and its impact on hospital revenue. The methodology section is well-detailed, explaining the study design and data analysis methods comprehensively. Results are presented logically with figures and tables supporting the findings. The discussion elaborates on the implications of inaccurate coding well, emphasizing the financial consequences and patient care.

 The manuscript provides valuable insights into the impact of inaccurate clinical coding on hospital finances. It has the potential to make a significant contribution to the field.

Is the work clearly and accurately presented and does it cite the current literature?

Yes

If applicable, is the statistical analysis and its interpretation appropriate?

Yes

Are all the source data underlying the results available to ensure full reproducibility?

Yes

Is the study design appropriate and is the work technically sound?

Yes

Are the conclusions drawn adequately supported by the results?

Yes

Are sufficient details of methods and analysis provided to allow replication by others?

Yes

Reviewer Expertise:

biomedical informatics

I confirm that I have read this submission and believe that I have an appropriate level of expertise to confirm that it is of an acceptable scientific standard.

# Reviewer report: Reviewer response for version 1

**DOI:** 10.5256/f1000research.163572.r310558

**Published:** 2024-08-07

**Authors:** Shankar Srinivasan

**Affiliations:** 1 Department of Health Informatics, Rutgers University New Brunswick, New Brunswick, New Jersey, USA

The article is very timely and impactful since it is appropriate to evaluate the current coding practices, their limitations and the costs they have on both patients and hospitals.   The author have used the appropriate data sources as also methodology for assessing their measures they had set out as being appropriate for the goals of  the project. They have also employed the appropriate statistical techniques (fairly straightforward ones given the nature of the project and its goals and which yielded appropriate results for an effective interpretation and reporting.

 Though the authors have used appropriate data for their analyses it would be great to further increase the data, perhaps from several more hospitals, so that the results may reveal some commonality amongst the hospital departments generating such errors. This will help towards selecting appropriate remedial measures to minimize such occurrences in future. Overall it is a novel project with lot of potential for further research.

Is the work clearly and accurately presented and does it cite the current literature?

Yes

If applicable, is the statistical analysis and its interpretation appropriate?

Yes

Are all the source data underlying the results available to ensure full reproducibility?

Yes

Is the study design appropriate and is the work technically sound?

Yes

Are the conclusions drawn adequately supported by the results?

Yes

Are sufficient details of methods and analysis provided to allow replication by others?

Yes

Reviewer Expertise:

Health Informatics, hospitalization outcomes research, health data analytics.

I confirm that I have read this submission and believe that I have an appropriate level of expertise to confirm that it is of an acceptable scientific standard.

# Response

**Published:** 2024-08-07

**Authors:** Salem Albagmi

**Affiliations:** Prince Sultan Military College of Health Sciences, Saudi Arabia

Thank you for your valuable insights on data, methodology, and statistical techniques. Your suggestion to expand the dataset is noted for a broader perspective. Understanding common trends can enhance remedial measures.

 Acknowledging the study's potential for further research is motivating. Continued exploration may lead to significant insights and practical solutions. Your feedback guides the study's future direction for impactful advancements in healthcare coding practices. Thank you for your insightful recommendations.
